# Prediction of maxillary canine impaction based on panoramic radiographs

**DOI:** 10.1002/cre2.246

**Published:** 2019-09-26

**Authors:** Raes Margot, Cadenas De Llano‐Pérula Maria, Alqerban Ali, Laenen Annouschka, Verdonck Anna, Willems Guy

**Affiliations:** ^1^ Department of Oral Health Sciences–Orthodontics, KU Leuven and Dentistry University Hospitals Leuven Leuven Belgium; ^2^ Department of Preventive Dental Sciences, College of Dentistry Prince Sattam Bin Abdulaziz University Al‐Kharj Saudi Arabia; ^3^ Interuniversity Institute for Biostatistics and statistical Bioinformatics KU Leuven and University Hasselt Belgium

**Keywords:** cuspid, impacted tooth, panoramic radiography, predictor

## Abstract

**Objectives:**

The objective of this article is to establish a large sample‐based prediction model for maxillary canine impaction based on linear and angular measurements on panoramic radiographs and to validate this model.

**Materials and methods:**

All patients with at least two panoramic radiographs taken between the ages of 7 and 14 years with an interval of minimum 1 year and maximum 3 years (T1 and T2) were selected from the Department of Oral Health Sciences, University Hospital Leuven database. Linear and angular measurements were performed at T1. From 2361 records, 572 patients with unilateral or bilateral canine impaction were selected at T1. Of those, 306 patients were still untreated at T2 and were used as study sample. To construct the prediction model, logistic regression analysis was used.

**Results:**

The parameters analyzed through backward selection procedure were canine to midline angle, canine to first premolar angle, canine cusp to midline distance, canine cusp to maxillary plane distance, sector, quadratic trends for continuous predictors, and all pairwise interactions. The final model was applied to calculate the likelihood of impaction and yielded an area under the curve equal to 0.783 (95% CI [0.742–0.823]). The cut‐off point was fixed on 0.342 with a sensitivity of 0.800 and a specificity of 0.598. The cross‐validated area under the curve was equal to 0.750 (95% CI [0.700, 0.799]).

**Conclusion:**

The prediction model based on the above mentioned parameters measured on panoramic radiographs is a valuable tool to decide between early intervention and regular follow‐up of impacted canines.

## 1 INTRODUCTION

The maxillary permanent canines are the most frequently impacted teeth, with exception of the third molars. This condition has a prevalence of 1–2.5%.[Ericson & Kurol, 1986; Thilander & Jakobsson, 1968] There could be a gender‐dependent tendency in canine impaction as it has been reported in literature that females are two to three times more affected than males.[Becker & Chaushu, 2015] There is no single or exclusive cause for maxillary canine impaction. Besides dental discrepancy or lack of space, presence of hard, and soft tissue pathologies are considered as etiological factors that can interfere with the normal path of maxillary canine eruption.[Alqerban, Jacobs, Fieuws, & Willems, 2015; Becker & Chaushu, 2015] A degree of autonomous correction and spontaneous eruption of impacted canines can be expected by eliminating the etiological factor.[Becker & Chaushu, 2015]

According to Becker et al., a correlation exists between lateral incisor anomalies and maxillary canine impaction, as the prevalence of impaction was greater when lateral incisors were developmentally missing, peg shaped, or hypoplastic.[Alqerban et al., 2015; Becker & Chaushu, 2015] The guidance theory proposes that these anomalies interfere with the guidance that lateral incisors give to the developing canines, leading to their palatal displacement.[Becker & Chaushu, 2015; Bishara et al., 1976] In contrast with this idea, according to the genetic theory, a palatally displaced maxillary canine is caused by genetic factors, and primary displacement of the tooth bud would be exclusively hereditary.[Bishara et al., 1976; Manne, Gandikota, Juvvadi, Rama, & Anche, 2012]

Early diagnosis is important and interceptive treatment of maxillary canine impaction is crucial because it reduces treatment costs and time, decreases risks of complications or adverse outcomes, and facilitates orthodontic mechanics.[Alqerban, Storms, Voet, Fieuws, & Willems, 2016] It is also of great importance to decrease the risk of lateral, and sometimes central, incisor root resorption.[Alqerban, Jacobs, Lambrechts, Loozen, & Willems, 2009] Extraction of the deciduous canines can encourage spontaneous eruption of the permanent canines.[Baccetti, Mucedero, Leonardi, & Cozza, 2009; Leonardi, Armi, Franchi, & Baccetti, 2004; Naoumova, Kürol, & Kjellberg, 2014; Naoumova & Kjellberg, 2018; Parkin, Benson, & Shah, 2009] Other complications may result if early intervention is not performed, such as pain, infection, cyst formation, ankylosis, internal or external resorption of the canine, and the adjacent teeth.[Alqerban, Jacobs, Lambrechts, et al., 2009; Bishara et al., 1976]

The background of the present study was based on a previous study performed by Alqerban et al.[Alqerban, Storms, et al., 2016] He suggested a prediction model based on the following parameters: the angle between the long axis of the canine and the first premolar (3^4), the perpendicular distance between the canine cusp and the midline (3c‐ML), and between the canine cusp and the maxillary plane (3c‐OP1).[Alqerban, Storms, et al., 2016] This approach could detect these three discriminating parameters and might differentiate between early intervention and regular follow‐up of canine impaction. However, the proposed protocol has not been validated on a larger population. Given this, the aims of the present study are to establish a new prediction model based on multiple linear and angular measurements on panoramic radiographs for the prediction of maxillary canine impaction of a larger sample and to validate the new and the existing prediction model.[Alqerban, Storms, et al., 2016]

## 2 MATERIALS AND METHODS

The study protocol was approved by the Medical Ethics Committee of the University Hospital Leuven with the registration number S60977.

### 2.1 Patient selection and data collection methods

The database of the Department of Oral Health Sciences, University Hospital Leuven was screened using the software Impax 6.5.5.1608 for panoramic images taken between March 2003 and October 2016. The search included all patients with at least two panoramic radiographs taken between the ages of 7 and 14 years with an interval of minimum 1 year and maximum 3 years (T1 and T2). Exclusion criteria were poor image quality and patients with pathology, such as syndromes, cleft lip and palate, and severe abnormalities. Patients with early extractions, orthodontic extrusion of the canine or canines that are already erupted on the first panoramic radiograph were excluded too.

**Figure 1:**
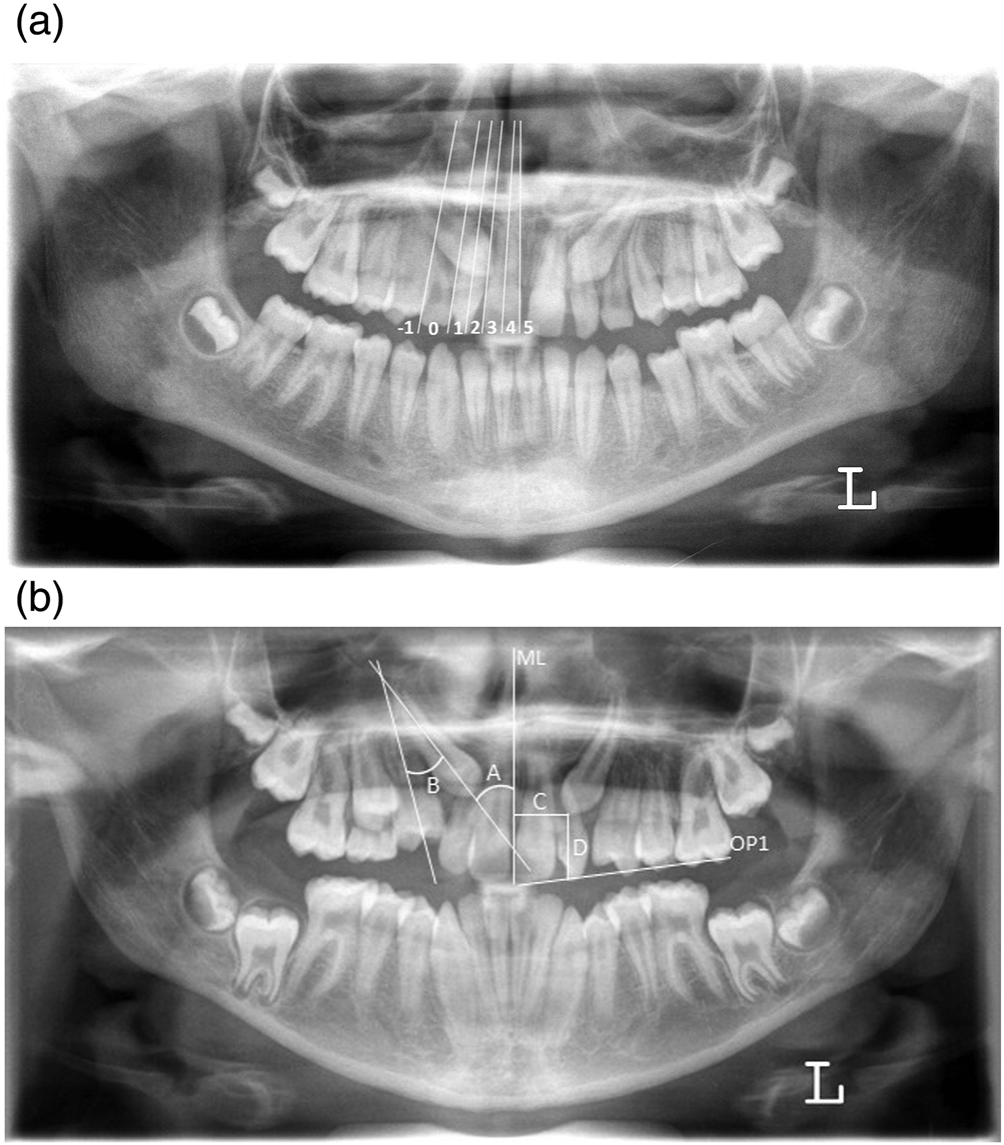
(a) Panoramic radiograph illustrating the sector of the canine. A modification of Ericson and Kurol's method was used. (b) Panoramic radiograph illustrating the angular measurements of the canine position in degrees, with (A) angle of maxillary canine to midline, (B) angle of maxillary canine to first premolar and linear measurements in millimeters with (C) canine cusp to midline distance, and (D) canine cusp to maxillary plane distance

Determination of impaction was made at T1 on the panoramic radiographs of the 2,361 subjects who met the inclusion criteria through the amount of canine overlap with the lateral incisor (sector) and the angle between the long axis of the canine and the midline (3^ML). A modification of Ericson and Kurol's method was used for scoring sector (Figure 1a).[Alqerban, Jacobs, Fieuws, & Willems, 2016; Ericson & Kurol, 1988a] A maxillary canine was considered to be impacted when the sector was greater than or equal to two and/or 3^ML was ≥15°.[Alqerban et al., 2014; Warford, Grandhi, & Tira, 2003] Five hundred seventy two patients were selected who showed a unilateral or bilateral maxillary canine impaction at T1. Of those, 306 patients were still untreated at T2.

For this study sample, the following parameters were collected: gender, date of birth, date of the panoramic radiographs, sector where the canine was positioned, 3^ML, 3^4, 3c‐ML, and 3c‐OP1. These linear and angular measurements (Figure 1b) were performed on both panoramic radiographs (T1 and T2) of the 306 subjects. For the angular measurements, the tooth axis was used. The midline was defined by the middle point between the two incisors and the anterior nasal spine. For the linear measurements, calibration was done by using a multiplication of the maxillary central incisor width at the contralateral side.[Alqerban, Storms, et al., 2016] The canine cusp to the midline or to the maxillary plane distance was measured perpendicular to each plane, respectively. The maxillary occlusal plane was determined by the incisal edge of the maxillary central incisor on the given side and the mesiobuccal cusp tip of the first maxillary molar.

### 2.2 Statistical analysis

All analyses were performed at the level of the tooth to quantify the performance of a new prediction model for impaction (T2) of previously impacted teeth (T1). Patients appear twice in the data set when both maxillary canines were impacted at T1. Logistic regression was used to construct the prediction model and estimate the model parameters. A backward selection procedure was applied, including all five selected parameter variables, quadratic trends for the four continuous parameters (3^ML, 3^4, 3c‐ML, and 3c‐OP1), and all pairwise interactions.

The area under the curve (AUC) was estimated as an indicator of discriminative value of the prediction model. To quantify the performance of the new model, a five‐fold cross validation was executed. In addition, validation of the existing prediction model was carried out.[Alqerban, Storms, et al., 2016]

Reliability analysis was performed. For 85 of the 306 patients of the original sample, all measurements (both elements, both T1 and T2) were repeated independently by the same observer. Additionally, a second investigator performed all measurements again for 30 patients. The intraclass correlation coefficients were determined as a quantification of the reliability of the measurements, taking values between zero and one, with values closer to one indicating higher reliability.

The data were statistically analyzed using SAS software (version 9.4 of the SAS System for Windows). To determine any significant differences the *p* values were set at.05.

## 3 RESULTS

The descriptive data of the sample have been listed in Table 1. On patient level, there were 118 subjects with a unilateral impacted maxillary canine and 188 with a bilateral maxillary canine impaction at T1. The mean age at T1 was 9.3 years (±1.32). On tooth level, 494 canines were listed as impacted at T1. Of these, 240 and 254 impactions of the right and left maxillary canine, respectively, were detected. The mean age at T2 was 11 years (±1.35). At T2, 40.69 % of the maxillary canines were still impacted.

**Table 1:** Descriptive information based on patient level and on tooth level at T1 (OPG1). Angles are shown in degrees and distances in millimeters. The values for the angular and linear measurements refer to all canines irrespective of side

Variable	Statistic	All
PATIENT LEVEL AT T1		
Total number of subjects	N	306
Gender		
Male	N	154
Female	N	152
Age at T1	Mean	9.3 (SD 1.32)
	Range	7;13
Number of subjects with impacted canines at T1		
Unilateral	N	118
Bilateral	N	188
TOOTH LEVEL AT T1		
Tooth	N	494
13	N	240
23	N	254
Canine† to first premolar† angle (degrees)	Mean	11.1 (SD 12.2)
3^4	Range	0.0;110
Canine cusp to midline^‡^ distance^¶^ (mm)	Mean	171.3 (SD 27.5)
3c‐ML	Range	88.9;271.2
Canine cusp to maxillary plane§ distance^¶^ (mm)	Mean	174.2 (SD 40.6)
3c‐OP1	Range	18.7;275.5
Canine† to midline‡ angle (degrees)	Mean	21.7 (SD 7.1)
3^ML	Range	2.4;77.8
Sector		
0	N	373
1	N	61
2	N	34
3	N	2

Abbreviation: SD, standard deviation.

† Tooth axis was used for canines and first premolars.

‡ The midline was defined by the middle between the two incisors and the anterior nasal spine.

§ The maxillary occlusal plane was determined by the incisal edge of the maxillary central incisor on the given side and the mesiobuccal cusp tip of the first permanent maxillary molar.

¶ The canine cuspid to the midline or maxillary plane distance was measured perpendicular to each other.

### 3.1 New prediction model and validation

Five possible predictor variables were selected (Table 1). With these parameters, a model was constructed excluding the observation with sector level 3. Table 2 shows the selected model variables with their *p* values. The variables with *p* > .05 were included in the model too because they are part of one of the interactions of variables that are significant (Table 2).

**Table 2:** Selected model variables with *P* values

Effect	*P* value
3^4	.0004*
3c‐ML	.9744
3c‐OP1	.5095
3^ML	.0549
Sector	.0441*
3^4. 3^4 ^†^	.0007*
3^4. 3c‐ML	.0256*
3^4. 3c‐OP1	.0003*
3^4. Sector	.0064*
3c‐OP1. Sector	.0368*

*Note.* Refer to table 1 for descriptions of symbols given in this table. The variables with *p* > 0.05 were included in the model because they are part of one of the interactions of variables that are significant.

†Point (.) means a multiplication of the given variables.

* *p* < .05.

**Figure 2:**
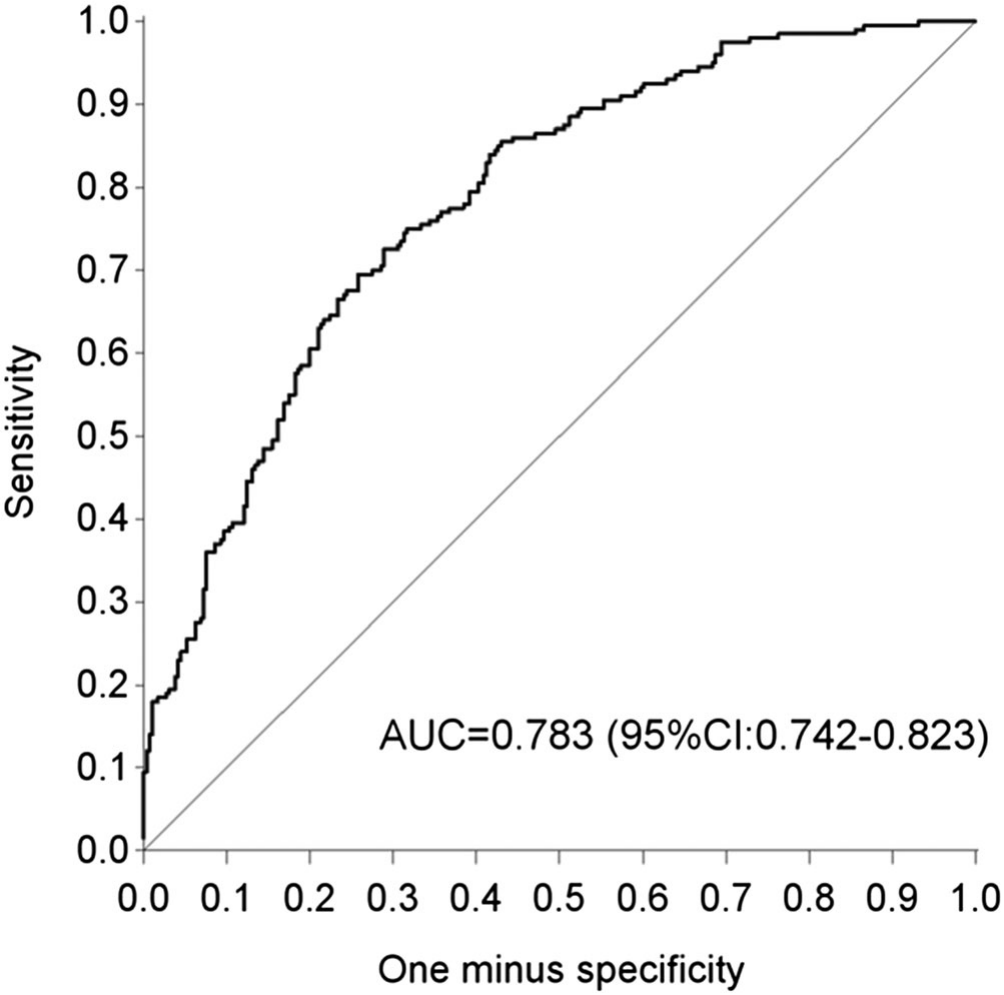
Receiver operating characteristic curve based on the multiple logistic regression analysis. It represents the sensitivity and (one minus) specificity of all possible classifications using different cut‐offs for the predicted probability of maxillary canine impaction. The optimal cut‐off point of probability is equal to.342 with a sensitivity of 80 % and a specificity of 59.8%

The final model was applied to calculate the likelihood of impaction yielding an AUC equal to 0.783 (95% CI [0.742–0.823]; Figure 2). The cross‐validated AUC was equal to 0.750 (95% CI [0.700, 0.799]), which implies a fair performance of the model. The cut‐off point of probability was fixed on.342 with a sensitivity of 80 % and a specificity of 59.8 %. A canine would be classified as impacted when the predicted probability of impaction (PI) exceeded.342. To calculate the PI, the weighted sum of the predictor values (=μ) must be determined from the multiple logistic regression model shown in Table 3, as follows (Table 3):

**Table 3:** Prediction model. To calculate the probability of impaction, the weighted sum of the predictor values (=μ), must be determined from the following multiple logistic regression model

Effect	Sector level	Parameter estimate	Standard error
Intercept		‐3.5494	3.0850
3^4		‐0.5533	0.1582
3c‐ML		0.0002	0.0066
3c‐OP1		0.0199	0.0124
3^ML		0.0430	0.0224
Sector	0	0.2681	2.7491
Sector	1	1.5664	2.9732
Sector	2	5.9506	3.2249
3^4. 3^4 ^†^		0.0025	0.0007
3^4. 3c‐ML		0.0013	0.0006
3^4. 3c‐OP1		0.0014	0.0004
3^4. Sector	0	‐0.0324	0.0704
3^4. Sector	1	0.0520	0.0768
3^4. Sector	2	0.1172	0.0787
3c‐OP1. Sector	0	‐0.0064	0.0122
3c‐OP1. Sector	1	‐0.0181	0.0136
3c‐OP1. Sector	2	‐0.0388	0.0164

*Note.* Refer to table 1 for descriptions of symbols given in this table.

†Point (.) means a multiplication of the given variables.

*PI* = exp(μ)/(1 + exp (μ))Where μ = ‐3.5494‐0.5533 (3^4) + 0.0002 (3c‐ML) + 0.0199 (3c‐OP1) + 0.0430 (3^ML) + 0.2681 (if *sector* = 0) + 1.5664 (if *sector* = 1) + 5.9506 (if *sector* = 2) + 0.0025 (3^4*3^4) + 0.0013 (3^4*3c‐ML) + 0.0014 (3^4*3c‐OP1)‐0.0324 [p3^4*(if *sector* = 0)] + 0.0520 [3^4*(if *sector* = 1)] + 0.1172 [3^4*(if *sector* = 2)]‐0.0064 [3c‐OP1*(if *sector* = 0)]‐0.0181 [3c‐OP1*(if *sector* = 1)]‐0.0388 [3c‐OP1*(if *sector* = 2)]

### 3.2 Validation of former prediction model

Validation of the former prediction model, using the new sample, resulted in a modest performance yielding an AUC of 0.594 (95% CI [0.544, 0.645]).[Alqerban, Storms, et al., 2016]

### 3.3 Reliability analysis

Very high intraclass correlation coefficients values were found, indicating very good to excellent inter‐rater and intra‐rater reliability of the measurements. The intra‐rater reliability with 95% confidence interval was 0.870 [0.840, 0.895], 0.920 [0.900, 0.935), 0.927 [0.909, 0.941], and 0.973 [0.966, 0.978] for 3^4, 3c‐ML, 3c‐OP1 and 3^ML respectively. The interrater reliability with 95% confidence interval was 0.954 [0.931, 0.970], 0.959 [0.938, 0.973], 0.943 [0.915, 0.963], and 0.984 [0.975, 0.989] for 3^4, 3c‐ML, 3c‐IP1, and 3^ML, respectively.

## 4 DISCUSSION

Impaction of the maxillary canine is a very common clinical manifestation. Early diagnosis of canine impaction is important due to the risk of root resorption of the neighboring permanent incisors, and therefore, it would be valuable to identify a new prediction model for the prediction of maxillary canine impaction. Over the years, numerous authors have already suggested various parameters for distinction of canine impaction.[Alqerban et al., 2015; Alqerban, Storms, et al., 2016; Chaushu, Chaushu, & Becker, 1999; Ericson & Kurol, 1987; Sajnani & King, 2012; Sambataro, Baccetti, Franchi, & Antonini, 2005] Despite the many publications in this area, associated factors remain a topic for discussion. For the determination of impaction, we used the sector and 3^ML. The sector has been proposed as the most important predictor for canine impaction.[Warford et al., 2003] Ericson and Kurol also proposed 3^ML as a powerful indicator of impaction.[Ericson & Kurol, 1988b] In addition, three other variables were selected based on the current literature. 3c‐OP1 was larger in patients with an impacted maxillary canine.[Ericson & Kurol, 1988b] 3^4 and 3c‐ML were also indicated as good discriminators.[Alqerban, Storms, et al., 2016] These findings supported the idea to use these parameters in the new prediction model.

Furthermore, 3^4 and sector were verified as independent variables, and interactions were found from the model fitting process (Table 2). They were incorporated in the final prediction model because they were significant at the *p* < .05 level or part of an interaction. Several studies have evaluated each parameter independently, but this is insufficient to accurately investigate such a complex dataset.[Al‐Nimri & Gharaibeh, 2005; Langberg & Peck, 2000] In the present study, the parameters were evaluated for quadratic trends and for pairwise interactions, which allows for reducing the extensiveness of the information while retaining valuable data about each measurement. Uribe et al. confirmed that research is more powerful when a multivariable data analysis is applied.[Uribe, Ransjö, & Westerlund, 2017] As mentioned in Materials and Methods section, the new model was constructed excluding the observations with Sector 3, allowing interactions of other variables with sector. This is due to the fact that model fitting would be unstable when interactions with sector are included, given that there are only two observations for Sector 3. Also, canines in Sector 3 are prone to be impacted, making the need for a prediction model unnecessary in those cases.[Warford et al., 2003]

The receiver operating characteristic curve shows the true positive rate against the false positive rate for the different possible cut‐off points of the present diagnostic tests (Figure 2). Any decrease in specificity will be accompanied by an increase in sensitivity. The discriminative ability of the present prediction model was 78.3 %. The cut‐off value was set to 0.342 to maximize the sensitivity. This value was accompanied by a sensitivity percentage of 80 %, which implies that 20 % of the non‐impaction predictions would be false. Furthermore, 59.8 % of the non‐impacted teeth have been correctly identified. So, this ensured that 40.2 % of the cases were false positive. This means that, in case it is decided to treat the impaction, the possibility of overtreatment should be taken into account.

In dentistry, panoramic radiographs are routinely taken for diagnosing and are therefore often present in patients' records. The methodology of the present study was to establish a screening system for impaction based on the available images, to allow early intervention of possibly impacted maxillary canines. Nevertheless, two‐dimensional radiographs demonstrate a number of well‐described limitations such as magnification, loss of information, overlapping, and distortion.[Holberg, Steinhäuser, Geis, & Rudzki‐Janson, 2005] Thus interpretations of measurements on panoramic radiographs may be affected if errors occur during radiographic imaging.[Henrique, Rondon, Carla, Pereira, & Crivelaro, 2014] Patient positioning errors are the most frequent type of error in panoramic radiography, and they cause radiographs with poor quality.[Dhillon et al., 2012; Henrique et al., 2014] Measurement errors due to head positioning effects are greater for the conventional panoramic radiographs when compared with three‐dimensional imaging.[Kitai et al., 2017] McKee et al. examined the effect of this patient positioning errors in panoramic radiography.[Mckee et al., 2001] They revealed that the majority of image angles from the deviating head positions were significantly different from the angles from the ideal head position.[Mckee et al., 2001] Maxillary teeth are unaffected by a right or left head rotation of 5° and are more sensitive to up or down head rotation of 5°.[Mckee et al., 2001] In general, relatively accurate assessment of angulations could be acquired using two‐dimensional radiography with occlusal plane variations of less than 10°.[Nikneshan, Sharafi, & Emadi, 2013] But the angular measurements on panoramic images should be interpreted with caution and with an understanding of patient positioning errors and the inherent image distortions.[Henrique et al., 2014; Mckee et al., 2001]

These problems can be solved by using three‐dimensional radiographs. However, especially in young children the as low as reasonably achievable principle applies here.[Oenning et al., 2018] Therefore, it remains important to perform measurements on commonly available panoramic images. Although cone‐beam computed tomography (CBCT) has earned its place for precise diagnostics related to impaction of the maxillary canines and the surrounding bone, linear and angular measurements can be precisely executed on panoramic radiographs.[Alqerban, Jacobs, Souza, & Willems, 2009; Alqerban, Jacobs, Fieuws, Nackaerts, & Willems, 2011; Alqerban et al., 2013; Alqerban et al., 2014; da Silva Santos et al., 2014; gao, lin, yan, Y tang, & chen, 2008; Holberg et al., 2005] Because of this, there would be no additional benefit to using CBCT for this study.[Stramotas, Geenty, Petocz, & Darendeliler, 2002; Volchansky, Cleaton‐Jones, Drummond, & Bonecker, 2006] Nevertheless, it seems that different diagnostic techniques, such as measurements on apical radiographs, render different results.[Alqerban, Jacobs, et al., 2016; Sambataro et al., 2005] For that reason, this prediction model may be useful in practices applying similar diagnostic and follow‐up strategies but should not be used as a standard protocol.

Another potential weakness of the present study is that it was a retrospective study. Therefore, it is important to interpret the results carefully in clinical settings. A prospective follow‐up of a potential impaction could give more power and can confirm its diagnosis. To meet this shortcoming both the old and the new formula were validated in this study. A five‐fold cross validation was performed to validate the new model. The cross‐validated AUC gives a reliable estimate of predictive accuracy of the model in a new sample. This to compensate for the over optimism. The new dataset was used to validate the old model, which gives us a rather weak AUC index of 0.594. A strength of the present study is the large sample size. Selecting at random one tooth out of two in the set of 188 patients with two impacted canines would have led to a smaller sample and hence, would have provided less power and would likely have led to a more parsimonious model. No bias is expected as a result of such a random selection.

Additional tools in the identification of canine impaction continue to play an important additional role such as the clinical experience of the practitioners and individual patient factors (age, gender). It is important to also include family history, visual inspection (deep bite, constricted maxilla, prolonged retention of the deciduous canine, lateral incisor anomalies), tactile clinical examination (absence of the canine bulge), and other radiographic signs (enlarged follicular sac, lack of resorption of the primary canine). These red flags need further investigation, for example by using the prediction formula. Furthermore, the choice of treatment depends on the age. A surgical exposure will not be performed to a maxillary canine at an age of 7 years.

Despite these limitations, determining a prediction model for maxillary canine impaction is clinically very important. The present prediction formula can help the practitioner to make an objective scientifically‐based decision and to predict presence of impaction based on the existing panoramic radiograph. To investigate this prediction model, a prospective study design is needed. And for more reliable measurements these variables should be studied using CBCT.

Alqerban states that CBCT could be a reliable diagnostic tool for detecting canine impaction. Knowing the shortcomings of panoramic radiographs, however they are still used routinely for pre‐orthodontic diagnostics in our country and that is why they are used in our study. Further, based on the panoramic image, it can be decided to take an additional CBCT. Additional parameters, such as arch length deficiency, that are associated with the etiology of impacted canines need further investigation.

## 5 CONCLUSION

This article presented a final prediction model based on radiological parameters measured on panoramic radiographs. The purpose is to sketch a clinically realistic scenario for orthodontists to contribute to anticipating on possible problems with the maxillary canines because panoramic images are routinely taken for pre‐orthodontic diagnosis. The model is useful to decide between early intervention and regular follow‐up of seemingly impacted canines. It can help to correct problems or point out the possible dangers of not treating them. Validation of the new model showed that detection of maxillary canine impaction on panoramic radiographs is a valuable tool.

## CONFLICT OF INTEREST

The authors certify no potential conflicts of interest with respect to the authorship and/or publication of this article.
